# Morbidity after elective surgery in patients on chronic dialysis: a systematic review and meta-analysis

**DOI:** 10.1186/s12882-021-02279-0

**Published:** 2021-03-18

**Authors:** Dharmenaan Palamuthusingam, Arun Nadarajah, David Wayne Johnson, Elaine Marie Pascoe, Carmel Marie Hawley, Magid Fahim

**Affiliations:** 1 Metro South Integrated Nephrology and Transplant Services, Logan Hospital, Armstrong Road & Loganlea Road, Meadowbrook, Queensland 4131 Australia; 2 Faculty of Medicine, University of Queensland, Armstrong Road & Loganlea Road, St Lucia, Queensland 4072 Australia; 3 School of Medicine, Griffith University, 68 University Dr, Meadowbrook, QLD 4131 Australia; 4 Department of Surgery, Sunshine Coast University Hospital, Doherty St, Birtinya, Queensland 4575 Australia; 5 Metro South and Integrated Nephrology and Transplant Services, Princess Alexandra Hospital, 199 Ipswich Road, Woolloongabba, Queensland 4074 Australia; 6 Translational Research Institute, Brisbane, Australia; 7 Centre for Health Services Research, University of Queensland, St Lucia, Queensland 4072 Australia

**Keywords:** Perioperative morbidity, Chronic dialysis, Surgery, Infection, End-stage kidney failure

## Abstract

**Background:**

Patients on chronic dialysis are at increased risk of postoperative mortality following elective surgery compared to patients with normal kidney function, but morbidity outcomes are less often reported. This study ascertains the excess odds of postoperative cardiovascular and infection related morbidity outcomes for patients on chronic dialysis.

**Methods:**

Systematic searches were performed using MEDLINE, Embase and the Cochrane Library to identify relevant studies published from inception to January 2020. Eligible studies reported postoperative morbidity outcomes in chronic dialysis and non-dialysis patients undergoing major non-transplant surgery. Risk of bias was assessed using the Newcastle-Ottawa Scale and the certainty of evidence was summarised using GRADE. Random effects meta-analyses were performed to derive summary odds estimates. Meta-regression and sensitivity analyses were performed to explore heterogeneity.

**Results:**

Forty-nine studies involving 10,513,934 patients with normal kidney function and 43,092 patients receiving chronic dialysis were included. Patients on chronic dialysis had increased unadjusted odds of postoperative cardiovascular and infectious complications within each surgical discipline. However, the excess odds of cardiovascular complications was attenuated when odds ratios were adjusted for age and comorbidities; myocardial infarction (general surgery, OR 1.83 95% 1.29–2.36) and stroke (general surgery, OR 0.95, 95%CI 0.84–1.06). The excess odds of infectious complications remained substantially higher for patients on chronic dialysis, particularly sepsis (general surgery, OR 2.42, 95%CI 2.12–2.72).

**Conclusion:**

Patients on chronic dialysis are at increased odds of both cardiovascular and infectious complications following elective surgery, with the excess odds of cardiovascular complications attributable to being on dialysis being highest among younger patients without comorbidities. However, further research is needed to better inform perioperative risk assessment.

**Supplementary Information:**

The online version contains supplementary material available at 10.1186/s12882-021-02279-0.

## Introduction

Advances in surgical techniques and perioperative care pathways have resulted in improved outcomes and facilitated access to surgery for increasingly complex patient cohorts, such as those with end-stage kidney disease (ESKD) [1]. A previous systematic review has shown that elective surgery in chronic dialysis patients is associated with a higher risk of postoperative mortality compared to patients with normal kidney function, which is at least partially due to a higher comorbid illness burden and older age [2–4]. Although death is the most serious complication, it is also an insensitive marker of cure or maintenance of function, and in fact qualitative research has shown that chronic dialysis patients prioritise maintenance of daily functional capacity over avoiding death itself [5, 6]. Therefore, accurate assessment of the risks of non-fatal postoperative outcomes may facilitate more meaningful patient engagement in shared decision-making regarding potential benefits and harms of surgery. Furthermore, perioperative risk assessment tools fail to include dialysis treatment as a risk factor, potentially leading to the use of unvalidated risk indices in this unique population.

The aims of this study were to ascertain the odds of non-fatal cardiovascular and infectious postoperative outcomes in patients receiving dialysis compared to patients with normal kidney function. A secondary aim was to explore the effects of age and non-kidney comorbidity on excess odds for these outcomes.

## Methods

This systematic review adhered to the Preferred Reporting Items for Systematic Reviews and Meta-analysis (PRISMA) [7] and Meta-analysis Of Observational Studies in Epidemiology (MOOSE) [8] checklists, with a protocol registered in PROSPERO (CRD42017076565). This paper focuses on the pre-specified secondary outcomes of the registered protocol pertaining to morbidity after elective surgery in patients on chronic dialysis. The search strategy and statistical analysis were adopted from this registered protocol [4].

### Search strategy

Without language restriction, MEDLINE, Embase and Cochrane Controlled Register of trials (CENTRAL) were searched for studies published until January 2020, using a combination of relevant keywords including surgery, dialysis, postoperative, perioperative, mortality, morbidity and their variants (Details of strategy in Supplementary Table 1, 2, 3, 4). Exploded MeSH terms for perioperative medicine and chronic dialysis patients were also used. Search terms were modified to correspond to the tree structure and descriptors of the two databases. Further studies were sought by manually searching reference lists of the relevant articles. In addition, tangential electronic exploration using links to related texts was also performed. Case-control studies, animal studies, opinion papers, case reports and editorials were excluded.

No existing reviews were identified in the Cochrane Database of Systematic Reviews (CDSR), Database of Abstracts of Reviews of Effects (DARE), NIHR Health Technology Assessment (NIHR HTA) programme and the National Institute for Health and Clinical Excellence (NICE) websites.

### Selection criteria

All cohort studies that measured and reported postoperative morbidity in adult (aged 18 years or older) chronic dialysis patients concurrently with patients who had normal kidney function were considered for inclusion. Normal kidney function was defined as a serum creatinine of less than 110 μmol/l or the absence of International Classification of Disease (ICD) coding of chronic kidney disease. All surgical disciplines were considered, including general, orthopaedic, cardiac, vascular and urology/gynaecological surgery. Studies where the proportion of urgent or emergent surgeries was less than 20% were included. Studies involving kidney transplantation, dialysis access surgery, and both endovascular and endoscopic procedures, were not included. Patients requiring chronic renal replacement therapy (CRRT) for an acute kidney injury undergoing surgery were not eligible for inclusion.

### Data extraction and outcome definition

Two researchers (DP and AN) independently screened all abstracts identified in the initial search to assess conformity with selection criteria. Disagreements were resolved by a third reviewer (MF). Data on the following characteristics were extracted independently by two investigators using a standard electronic data extraction form: type of surgery, numbers of dialysis patients, number of patients with normal kidney function, location (defined as Asia-Pacific, Europe and North America), summary statistics for patient baseline characteristics (including cardiovascular disease, peripheral vascular disease, diabetes mellitus, hypertension and smoking status), frequency of postoperative outcomes, and adjusted odds ratios where available.

The primary outcomes were postoperative myocardial infarction, stroke, surgical site infections (both superficial and deep), sepsis and pneumonia, defined as either within 30-days or within the same hospitalisation as the index surgery. Secondary outcomes were postoperative packed red cell transfusion, thromboembolic events and unplanned return to theatre. The definition used by a given study for each complication, if present, was noted at the time of data extraction. Furthermore, severity of complications, measured using the Clavien-Dindo Classification were recorded if available. The Clavien-Dindo classification is a scale from 1 to 5 measuring the implications of a post-operative complication on a patient’s treatment course and outcome [9]. Grade 1 refers to any deviation to the usual postoperative course, grade 2 refers to the need for complication-specific pharmacotherapy, grade 3 refers to the need for endoscopic or radiological intervention, grade 4 refers to the need for intensive care admission, and grade 5 refers to death arising from the post-operative complication.

Two independent reviewers assessed the methodological quality of each study using the Newcastle-Ottawa Scale (NOS), which employs a star system to evaluate the selection of the study groups (0–4 stars), comparability of the groups (0–2 stars), and ascertainment of the outcome of interest (0–3 stars) [10]. The GRADE (grading of recommendations assessment, development, and evaluation) approach was used to assess the certainty of evidence for each outcome [11]. The certainty of evidence was classified into one of four categories; high, moderate, low and very low (Supplementary Table 6).

### Statistical analysis

For all outcomes, an unadjusted odds ratio [OR] and 95% CI were calculated using the number of events in each group. Summary estimates were calculated using inverse variance weighted random effects meta-analysis [4]. Both individual study and summary odds estimates were displayed in forest plots by each surgical type. Surgical types were not combined in any analyses owing to clinical heterogeneity. Adjusted OR and 95% CI were recorded if studies performed a multivariable analysis, adjusting for age as a minimum.

To assess the relationship between the unadjusted effect size and important study level covariates, meta-regression was performed using the random effects model with two categories of predictor variables: study characteristics, including study quality (as per NOS), single versus multicentre cohorts, continents, study duration, and single procedure studies versus composite procedures, and patient characteristics, including age and relative prevalence of diabetes mellitus or ischemic heart disease among dialysis patients compared to non-dialysis patients, including age and pre-operative co-morbidity burden (ischemic heart disease and diabetes) [4].

L’Abbé plots were generated to identify studies responsible for divergent results [12]. Inter-rater reliability of study selection was assessed using Cohen’s kappa. A funnel plot and Egger’s test for funnel plot asymmetry were used to assess publication bias. Heterogeneity was assessed using *I*^*2*^ [13].

Statistical analysis was performed with Stata 14.0 for Windows. Statistical significance was defined as a two-sided *p*-value < 0.05.

## Results

### Study selection and characteristics

**Fig. 1:**
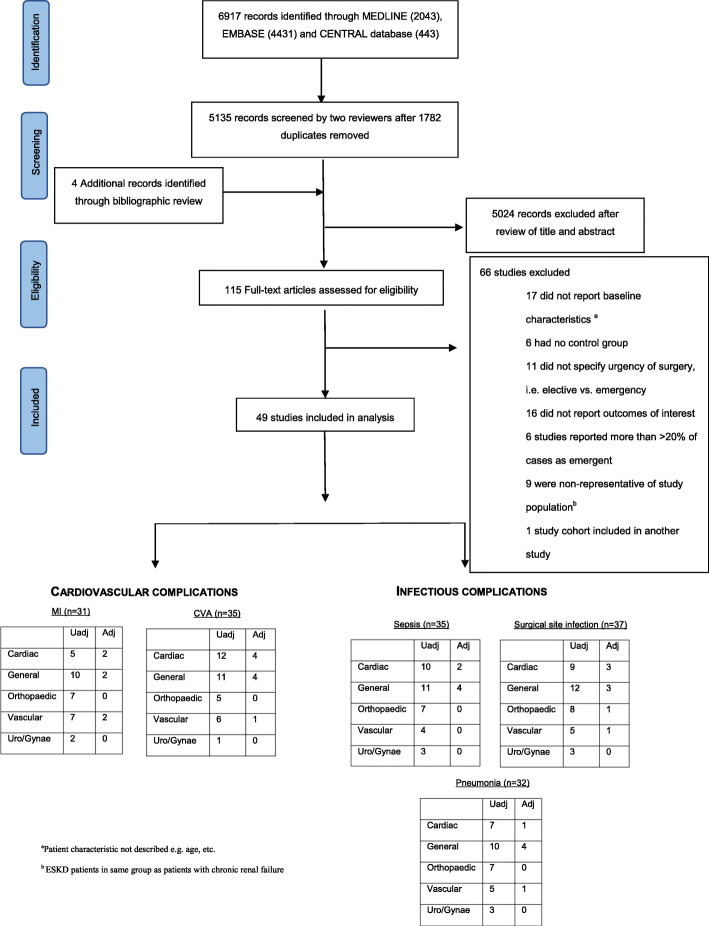
PRISMA flow diagram of study selection

In total, 5,135 abstracts were reviewed, from which 115 full-text articles were retrieved and evaluated (Fig. 1). Forty-nine studies, involving 10,513,934 patients with normal kidney function and 43,092 chronic dialysis patients, satisfied the inclusion criteria (Table 1: Summary of baseline characteristics of studies included). The definition of chronic dialysis varied across studies, with 22 studies using registry-based definitions, six using International Classification of Disease Coding (ICD) and the remaining studies confirming chronic dialysis status by medical chart reviews. Non-emergent cardiac surgery was the most commonly reported type of surgery (28%) [14–27], followed by general surgery (24%) [2, 28–38], orthopaedic surgery (22%) [39–49], vascular surgery (16%) [50–56], and urologic/gynaecologic surgery (8%) [57–60]. Twenty-six of the 49 studies assessed a single surgical procedure [18–20, 22, 24–26, 32, 34, 36–39, 41–43, 45, 48–50, 53, 54, 56–59], while the remaining 23 studies examined a mixture of discipline-specific surgical interventions.

**Table 1:** Baseline characteristics of included studies

Author	Country	Type of Surgery	Dialysis type	Total number of patients in study (n)		Mean age years (SD) or Median age [IQR]		Ischemic heart disease (%)		Diabetes (%)		Outcomes reported
				Normal kidney function	Dialysis patients	Normal kidney function	Dialysis patients	Normal kidney function	Dialysis patients	Normal kidney function	Dialysis patients	
**Cardiac Surgery**												
Al Sarraf, 2011	Ireland	Coronary artery bypass graft & Valve	Unspecified	3,276	45	63.2 (10.2)	62 (12.3)	2,763 (84.3)	36 (80)	560 (17.1)	10 (22.2)	Stroke, Sepsis, Pneumonia, Return to Operating theatre, Blood transfusion
Charytan, 2007	USA	Coronary artery bypass graft & Valve	Both	77,323	635	66.1^a^	62.9^a^	14,619 (18.9)	253 (39.8)	23,208 (30.0)	367 (57.8)	Stroke, Sepsis, Pneumonia,
Chikwe,2010	USA	Coronary artery bypass graft	Unspecified	2,803	96	65.6 (0.7)	63.3 (10.7)	1,398 (49.9)	52 (54.2)	1,092 (38.9)	69 (71.9)	Myocardial infarction, Stroke, Sepsis, Surgical site infection, Pneumonia, Return to Operating theatre
Cooper, 2006	USA	Coronary artery bypass graft & Valve	Unspecified	104,880	7,152	58.9a	63a	9,439 (8.9)	1,595 (22.3)	31,464 (30)	4,363 (61.0)	Stroke, Sepsis, Surgical site infection, Return to Operating theatre
Fukushima, 2005	Japan	Coronary artery bypass graft	HD	451	20	67.2 (8)	63.9 (8)	451 (100)	20 (100)	243 (53.9)	13 (65)	Stroke, Pneumonia
Griffin, 2019	USA	Coronary artery bypass and valve	HD	1,416	35	62.6 (14.3)	58.8 (13.0)	1,416 (100)	35 (100)	434 (30.7)	22 (62.9)	Surgical site infection, Sepsis, Pneumonia, Stroke,
Kan, 2004	Taiwan	Coronary artery bypass graft	Both	69	23	63.8 (11.2)	63.8 (9.9)	23 (33.3)	23 (100)	34 (49.3)	15 (65.2)	Sepsis, Surgical site infection, Return to Operating theatre
Murai, 2007	Japan	Coronary artery bypass graft	HD	60	39	67.2 (7.9)	63.2 (10.2)	60 (100)	39 (100)	NR	NR	Blood transfusion
Rahmanian, 2008	USA	Coronary artery bypass graft	Unspecified	6,449	245	63.9 (13.8)	61.3 (13.2)	1,548 (30.1)	81 (33.1)	1,548 (24.0)	104 (42.4)	Myocardial infarction, Stroke, Sepsis, Surgical site infection, Return to Operating theatre, Blood transfusion
Raza, 2017	USA	Valve surgery	HD	199	144	58^a^	55^a^	NR	NR	NR	NR	Myocardial infarction, Stroke, Sepsis, Return to Operating theatre
Yamauchi, 2012	Japan	Coronary artery bypass graft	HD	18,387	1300	68.7 (9.4)	65.4 (9.2)	NR	NR	8,826 (48.0)	854 (65.7)	Stroke, Sepsis, Surgical site infection, Pneumonia, Return to Operating theatre, DVT/PE, Blood transfusion
Wong, 2003	Canada	Coronary artery bypass graft	Unspecified	70	35	64 (11)	64 (11)	32 (45.7)	14 (40)	31 (44.3)	8 (22.9)	Myocardial infarction, Stroke, Surgical site infection, Return to Operating theatre, Blood transfusion
Vasileva, 2014	USA	Cardiac Valve surgery	Unspecified	85,083	1,480	63 [52–72]	55 [45–65]	NR	NR	10,295 (12.1)	463 (31.3)	Stroke, Surgical site infection, Pneumonia, Return to Operating theatre, Blood transfusion
Thourani, 2012	USA	Coronary artery bypass graft and Valve	Haemodialysis	5,084	224	61 (14.8)	54 (14.0)	798 (15.7)	46 (20.5)	933 (18.4)	87 (38.8)	Myocardial infarction, Stroke, Sepsis, Surgical site infection, Pneumonia, Return to Operating theatre
**General Surgery**												
Andalib, 2016	USA	Bariatric procedures	Both	113,677	234	44.7 (11.6)	47.3 (10.4)	154 (0.1)	1 (0.4)	30,602 (26.9)	112 (47.9)	Myocardial infarction, Stroke, Sepsis, Surgical site infection, Pneumonia, Return to Operating theatre, DVT/PE, Blood transfusion
Barbas, 2014	USA	Hepatobiliary	Both	27,275	101	62 [53–71]	60 [53–68]	2,204 (8.1)	17 (16.8)	5,431 (19.9)	48 (47.5)	Myocardial infarction, Stroke, Sepsis, Surgical site infection, Pneumonia, Return to Operating theatre, DVT/PE
Cherng, 2013	Taiwan	General	Both	8,937	8,937	65^a^	65.5^a^	3,965 (44.4)	3,930 (43.9)	4,628 (51.8)	4,589 (51.3)	Myocardial infarction, Stroke, Sepsis, Surgical site infection, Pneumonia
Cloyd, 2014	USA	General	HD	24,110	149	61.3 (15.5)	62.3 (13.4)	NR	NR	NR	NR	Myocardial infarction, Stroke, Sepsis, Surgical site infection, Pneumonia, DVT/PE
Ekici, 2009	Turkey	Laparoscopic Cholecystectomy	PD	33	11	45.6 (11)	44.2 (9.1)	NR	NR	NR	NR	Sepsis, Surgical site infection, Blood transfusion
Gajdos, 2013	USA	General surgery	Both	164,094	1,506	55.4 (16.8)	59.6 (15.1)	1,590 (1.0)	100 (6.6)	26,764 (16.3)	583 (38.7)	Myocardial infarction, Stroke, Surgical site infection, Pneumonia, Return to Operating theatre, DVT/PE
Hu, 2015	USA	Colorectal Surgery (General)	Unspecified	42,138	265	70 [31–90]	67 [19–90]	NR	NR	131 (49.4)	7760 (18.4)	Myocardial infarction, Stroke, Sepsis, Surgical site infection, Pneumonia, Return to Operating theatre, DVT/PE, Blood transfusion
Manrique, 2017	Taiwan	Skin grafting for head and neck reconstruction	Unspecified	841	85	55.9 (10.0)	56.3 (10.5)	NR	NR	139 (16.5)	52 (61.2)	Myocardial infarction, Stroke, Sepsis, Surgical site infection, Pneumonia, DVT/PE
Montgomery, 2019	USA	Bariatric surgery	Unspecified	417,403	1,244	44.2 [35.7–53.3]	49.0 [41.6–55.8]	12,497 (3.0)	199 (16.0)	109,509 (26.2)	690 (55.5)	Myocardial infarction, stroke, pneumonia, DVT/PE, Surgical site infection, Sepsis, Blood transfusion, Return to Operating theatre
Rao, 2014	USA	Cholecystectomy	Unspecified	80,483	512	48.9 (17.4)	59.8 (14.9)	563 (0.7)	26 (0.1)	8,853 (11.0)	235 (45.9)	Myocardial infarction, Stroke, Sepsis, Surgical site infection, Pneumonia, Return to Operating theatre, DVT/PE, Blood transfusion
Schneider, 2009	USA	General	Unspecified	108	54	59.8^a^	59.3^a^	NR	NR	NR	NR	Stroke, Sepsis, Surgical site infection
Tam, 2015	USA	Ventral hernia (General)	Unspecified	90,993	700	53.5 (14.5)	56.7 (13.5)	7,097 (7.8)	160 (22.9)	12,189 (13.4)	214 (30.6)	Myocardial infarction, Stroke, Sepsis, Surgical site infection, Pneumonia, Return to Operating theatre, DVT/PE, Blood transfusion
**Orthopaedic Surgery**												
Cancienne, 2019	USA	Shoulder arthroplasty	Both	3,675	1,225	67.5^a^	67.5^a^	2,570 (69.9)	881 (71.9)	2,766 (75.3)	922 (75.3)	Sepsis, Return to Operating theatre
Chikuda, 2012	Japan	Spinal surgery	HD	50,779	869	62.3 (15.6)	64.3 (8.2)	2,597 (5.1)	126 (14.5)	6252 (12.3)	166 (19.1)	Myocardial infarction, Stroke, Sepsis, Surgical site infection, Pneumonia, DVT/PE
Chung, 2017	USA	Spinal surgery	Unspecified	2,522,594	1,834	59.9 (14.7)	64.2 (11.6)	48,814 (1.9)	273 (14.9)	421,178 (16.7)	907 (49.5)	Myocardial infarction, Stroke, Sepsis, Pneumonia, DVT/PE, Blood transfusion
Hickson, 2018	USA	Hip arthroplasty	Both	1,508	377	78 [68–85]	77 [67–84]	NR	NR	648 (43.0)	162 (43.0)	Surgical site infection, Pneumonia, DVT/PE, Myocardial infarction, Sepsis
Inoue, 2018	Japan	Spinal Surgery	Unspecified	863	90	72.3 [30–92]	69.9 [48–68]	109 (12.6)	16 (17.8)	201 (23.3)	27 (30)	Surgical site infection, Myocardial infarction, Stroke, Blood transfusion
Lin, 2019	Taiwan	Hip Hemiarthroplasty	Unspecified	19,954	1,311	73.5 ± 11.2	70.7 ± 8.8	3,913 (19.6)	466 (35.5)	NR	NR	Surgical site infection, Pneumonia, Blood transfusion, Return to Operating theatre
Lizaur-Utrilla, 2016	Spain	Arthroplasty	HD	30	15	70.1 [58–72]	69.3 [56–72]	8 (26.7)	4 (26.7)	7 (23.3)	6 (40)	Myocardial infarction, Sepsis, Surgical site infection, Pneumonia, DVT/PE, Blood transfusion
Ottesen, 2018	USA	Spinal surgery	Unspecified	173,311	467	57.0 (5.5)	63 (7.0)	NR	NR	NR	NR	Myocardial infarction, Stroke, Sepsis, Surgical site infection, Pneumonia, Return to Operating theatre, DVT/PE
Ottesen, 2018	USA	Total knee arthroplasty	Unspecified	163,560	250	67.0 (7.0)	68.0 (7.0)	NR	NR	NR	NR	Myocardial infarction, Stroke, Sepsis, Surgical site infection, Pneumonia, Return to Operating theatre, DVT/PE
Ponumsamy, 2015	USA	Arthroplasty	Unspecified	6,186,475	2,934	66.8 (0.1)	66.7 (0.6)	NR	NR	NR	NR	Surgical site infection, Blood transfusion
Yu, 2011	Taiwan	Spinal surgery (Orthopaedic)	HD	34	43	61.5 (8.1)	62.5 (8.1)	NR	NR	3 (8.8)	7 (16.3)	Sepsis, Return to Operating theatre, Blood transfusion
**Vascular surgery**												
Ambur, 2019	USA	Lower extremity bypass	Both	6,101	550	68.2 (11.7)	66.4 (10.7)	188 (3.0)	42 (7.6)	2,924 (47.9)	349 (63.5)	Myocardial infarction, Return to Operating theatre
Balceniuk, 2019	USA	Distal lower extremity bypass	Both	12,006	1,014	68.4^a^	67.3^a^	209 (2.0)	29 (3.0)	5501 (57.0)	784 (81.0)	Myocardial infarction, Stroke
Gajdos, 2013	USA	Vascular procedures involving both peripheral arteries, carotid and aorta.	Both	34,813	1,409	70.3 (10.8)	64.3 (13.8)	7,129 (20.5)	335 (23.8)	9,029 (25.9)	726 (51.5)	Myocardial infarction, Stroke, Sepsis, Surgical site infection, Return to Operating theatre, DVT/PE
Hibino, 2016	Japan	Abdominal aortic aneurysm repair (Vascular)	HD	679	21	65.7 (13)	63.5 (15.5)	NR	NR	79 (11.6)	9 (42.9)	Stroke, Sepsis, Surgical site infection, Pneumonia, Return to Operating theatre, Blood transfusion
Hickson, 2018	USA	Lower extremity amputation	Unspecified	5,302	1,166	68.0 (11.0)	65.0 (7.5)	NR	NR	3,085 (58.2)	882 (75.6)	Myocardial infarction, Sepsis, Surgical site infection, Pneumonia
Lantis, 2001	USA	Infrainguinal bypass	Unspecified	481	60	69^a^	63.0^a^	241 (50.1)	38 (63.3)	212 (44.1)	50 (83.3)	Myocardial infarction,
O Hare 2003	USA	Non-traumatic lower limb amputation	Unspecified	11,051	1,110	68^a^	68^a^	884 (8.0)	189 (17.0)	5,747 (52.0)	799 (72.0)	Myocardial infarction, Stroke, Sepsis, Pneumonia, DVT/PE, Blood transfusion
Rao, 2017	USA	Infrainguinal bypass (Vascular)	Both	33,318	1,623	66.7 (11.0)	66.0 (10.7)	433 (1.3)	73 (4.5)	12,228 (36.7)	1,034 (63.7)	Myocardial infarction, Stroke, Sepsis, Surgical site infection, Pneumonia, Return to Operating theatre, DVT/PE
**Urology/gynaecology surgery**												
Fornara, 1998	Germany	Laparoscopic Nephrectomy	HD	20	19	38 [24–70]	45 [20–73]	NR	NR	NR	NR	Myocardial infarction, Sepsis, Pneumonia, Blood transfusion
May, 2018	USA	Laprascopic nephrectomy	Both	7,870	445	55	55	56 (0.7)	14 (3.1)	1,637 (20.8)	141 (31.7)	Surgical site infections, Sepsis, Pneumonia, DVT/PE, Blood transfusion
Schmitges, 2012	USA	Nephrectomy (Urologic/Gynaecologic)	Unspecified	3,764	941	59 (12.8)	59.1 (12.7)	NR	NR	NR	NR	Surgical site infection, Blood transfusion
Yamashita, 2012	Japan	Nephrectomy (Urologic/Gynaecologic)	Both	104	38	61.9 (14.1)	58.3 (11.4)	NR	NR	NR	NR	Sepsis, Surgical site infection, Pneumonia

^a^Standard deviation or interquartile range not provided

Thirty-seven studies failed to indicate dialysis modality, ten studies specifically examined haemodialysis patients only [18, 20, 22, 23, 26, 31, 33, 39, 43, 50, 57], and two studies looked at peritoneal dialysis patients separately [32, 49].

Of the 49 studies, 19 reported findings from a single centre [14, 16, 18–23, 25, 32, 35, 41, 43, 47, 50, 51, 57, 59, 61] and only three collected data prospectively [14, 18, 43]. Twenty-three studies extracted information from existing data registries while the remaining extracted information from re-examined health records. Thirty-three studies were reported from North America [2, 15–17, 21–24, 27–29, 31, 33–37, 40, 42, 44–46, 49, 51–56, 58, 60, 62], ten from Asia [18–20, 26, 30, 38, 39, 43, 47, 48, 50, 59] and four from Europe [14, 32, 41, 57]. Thirty-four studies were published after 2010 [2, 14, 16, 22–24, 27–31, 33, 34, 36–50, 53–56, 58–60, 62].

All 49 studies reported age and gender, but comorbidities were less consistently described, with 37 (76%) studies reporting the prevalence of diabetes mellitus [2, 14–19, 21, 23–30, 33, 34, 36–41, 43, 46, 47, 49–54, 60, 62], 32 (65%) reporting ischemic heart disease (IHD) [2, 14–21, 23, 25, 27–30, 34, 36–41, 46–49, 51–54, 60, 62], 20 (41%) reporting smoking status [17, 19, 23, 25, 26, 28, 29, 33–35, 37, 38, 46, 49–52, 54, 60–62], and 14 (29%) studies reporting all three comorbidities [19, 25, 28, 29, 33, 37, 46, 49, 51, 52, 54, 60, 63].

#### Cardiovascular complications

##### Myocardial infarction

**Fig. 2:**
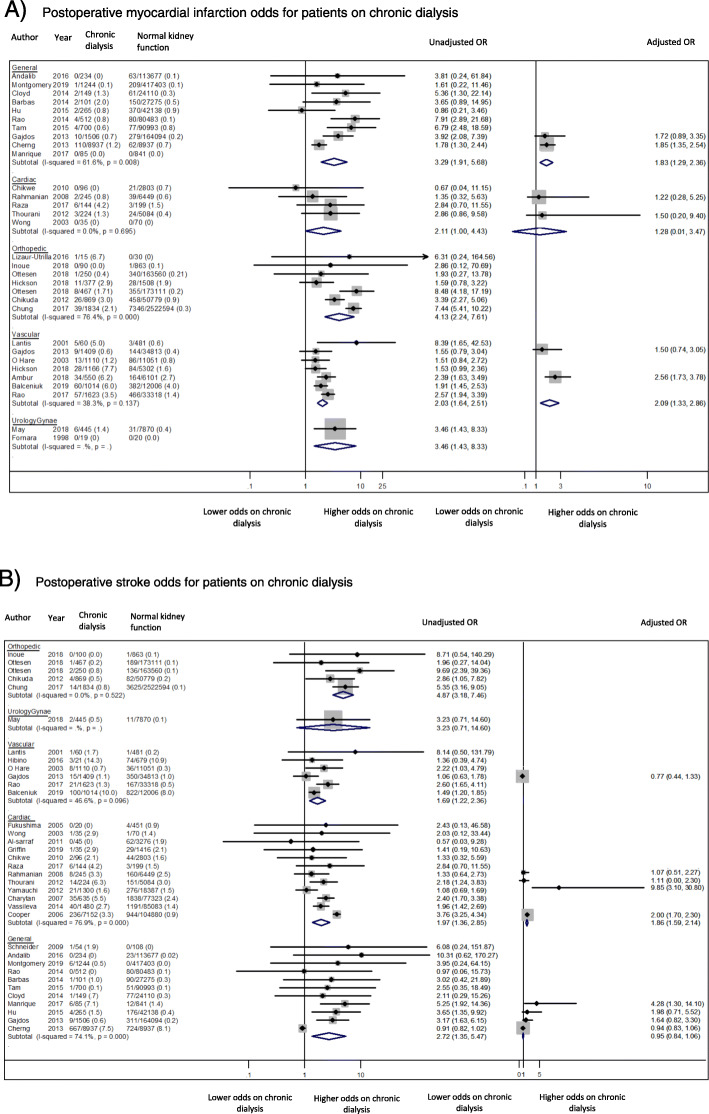
Cardiovascular Complications. **a** Postoperative myocardial infarction odds for patients on chronic dialysis. **b** Postoperative stroke odds for patients on chronic dialysis

Myocardial infarction was reported in 31 studies involving 25,775 patients receiving chronic dialysis and 4,008,163 patients with normal kidney function. Studies in general surgery most frequently reported myocardial infarction as a complication (10 of 12 studies). The incidence of postoperative myocardial infarction across all individual studies ranged from 0 to 6.7% in dialysis patients and 0–4.0% in patients with normal kidney function. The unadjusted odds of myocardial infarction was higher for chronic dialysis patients than for patients with normal kidney function in all surgical specialities, with the lower bounds of the 95% CI of the relative risk estimates for each surgical discipline equal to or greater than 1.0 (Fig. 2a). The highest odds was following orthopaedic surgery (7 studies, 3902 dialysis patients, OR 4.13, 95% CI 2.24–7.61, I^2^ = 76.4%, *p* < 0.001, moderate certainty evidence). Adjusted odds ratio estimates, with age as a minimum covariate, was reported by 5 studies in which the excess odds was attenuated but remained high following general surgery (2 studies, 10,443 dialysis patients, OR 1.83 95% 1.29–2.36, I^2^ = 0.0, *P* = 0.852, low certainty evidence).

##### Stroke

Thirty-five studies reported postoperative stroke, involving 34,400 chronic dialysis patients and 4,286,805 patients with normal kidney function. Stroke was most frequently reported by studies in cardiac surgery (12 of 14 studies), followed by studies in general surgery (11 of 12 studies). The incidence of stroke across all thirty individual studies ranged from 0 to 14.3% in chronic dialysis patients, and 0–10.9% in patients with normal kidney function. The median reported incidence of stroke in chronic dialysis patients by sub-specialty was highest following cardiac surgery at 2.9%, compared to 1.5% in their non-dialysis counterparts. Studies did not differentiate between off-pump coronary artery bypass surgery and conventional coronary artery bypass. Only one study involving urologic or gynaecologic surgery reported the incidence of stroke. The unadjusted odds ratio of stroke was considerably higher for patients receiving chronic dialysis compared to patients with normal kidney function in all surgical disciplines (Fig. 2b). The highest odds observed was following orthopaedic surgery (5 studies, 3,520 dialysis patients, OR 4.87, 95% CI 3.18–7.46, I^2^ = 0.0%, p for heterogeneity 0.52, moderate certainty evidence). Adjusted odds ratio estimates were provided by 9 studies. The summary odds risk estimate remained elevated following cardiac surgery (4 studies, 8921 dialysis patients, OR 1.86, 95%CI 1.59–2.14, I^2^ = 56.2%, *p* = 0.08, low certainty evidence) but was not significantly different from that of patients with normal kidney function following general surgery (4 studies, 10,793 dialysis patients, OR 0.95, 95%CI 0.84–1.06, I^2^ = 0.00, *p* = 0.40, low certainty evidence).

#### Infectious complications

##### Sepsis

**Fig. 3:**
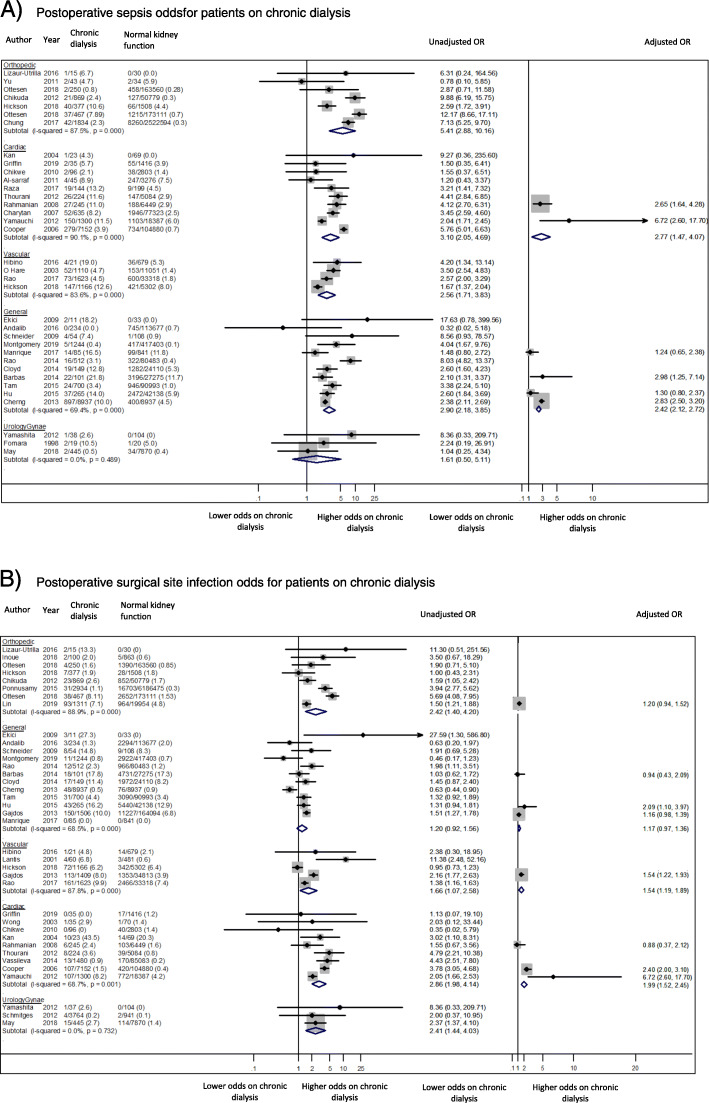
Postoperative Infectious Complications. **a** Postoperative sepsis odds for patients on chronic dialysis. **b** Postoperative surgical site infection odds for patients on chronic dialysis

Sepsis was reported in 35 studies, involving 3,996,044 patients with normal kidney function and 30,468 dialysis patients, and was most frequently reported following general surgery (11 of 12 studies). Across all studies, the incidences of sepsis ranged from 0 to 21.8% in dialysis-dependent patients and 0–11.8% in patients with normal kidney function. When comparing incidence rates between specialities, the highest median reported rate of sepsis in dialysis patients was 10.0% after general surgery. Meta-analysis showed that being on chronic dialysis was associated with an increased odds of developing postoperative sepsis, irrespective of surgical discipline (Fig. 3a). The highest excess odds was seen following orthopaedic surgery (7 studies, 3,855 dialysis patients, OR 5.41, 95%CI 2.88–10.16, I^2^ = 87.5% p for heterogeneity < 0.001, moderate certainty evidence). Summary odds ratio estimates from the adjusted results attenuated the excess odds (2 cardiac surgery studies, 1,545 dialysis patients, OR 2.77, 95% CI 1.47–4.07, I^2^ = 7.7%, *p* = 0.30, low certainty evidence; 4 general surgery studies, 9388 dialysis patients, OR 2.42, 95% CI 2.12–2.72, I^2^ = 85.3% *p* < 0.001, low certainty evidence).

##### Surgical site infection

Thirty-seven studies involving 7,877,144 patients with normal kidney function and 36,414 patients receiving chronic dialysis reported surgical site infections. The incidences of surgical site infections across the all studies ranged from 0 to 43.5% in dialysis patients and 0–20.3% in patients with normal kidney function. Compared to other specialties, the highest median incidence of surgical site infections for chronic dialysis patients was 7.2% following general surgery. The odds ratio of surgical site infection was higher among patients receiving chronic dialysis compared to patients with normal kidney function after all types of surgery except following urologic and gynaecologic procedures (Fig. 3b). There was almost a 3-fold increased excess odds of surgical site infections following cardiac surgery (9 studies, 10,590 dialysis patients, OR 2.86, 95% CI 1.98–4.14, I^2^ = 68.7% p for heterogeneity < 0.001, moderate certainty evidence). Meta-analysis of adjusted odds ratios for cardiac surgery attenuated the heightened odds in dialysis patients (3 cardiac surgery studies, 8,697 dialysis patients, OR 1.99, 95% CI 1.52–2.45, I^2^ = 79.6, *p* = 0.007, low certainty evidence).

##### Pneumonia

Thirty-two studies involving 4,185,864 patients with normal kidney function and 28,426 dialysis-dependent patients reported postoperative pneumonia. Ten of the 12 studies in general surgery reported pneumonia as a complication. The incidences of pneumonia across all studies ranged from 0 to 52.4% in dialysis patients and 0–13.7% in patients with normal kidney function. The postoperative odds ratio of developing postoperative pneumonia was higher in chronic dialysis patients compared to patients with normal kidney function across all surgical disciplines (Supplementary Figure 1). Adjusted odds ratios were provided by 6 studies. The excess odds remained elevated following general surgery (4 general surgery studies, 10,793 chronic dialysis patients, OR 1.54, 95% CI 1.37–1.71, I^2^ = 51.9 *p* = 0.10, moderate certainty evidence).

##### Other surgical outcomes

Patients receiving chronic dialysis were also at higher odds of other non-fatal surgical outcomes compared to patients with normal kidney function, including unplanned return to theatre (highest following general surgery; 7 studies 4,562 dialysis patients, OR 2.75, 95%CI 1.98–3.81, I^2^ = 95.2%, *p* = 0.001, low certainty evidence), blood transfusion requirement (highest following cardiac surgery; 6 studies, 3,144 patients, OR 4.23, 95%CI 2.80–6.37, I^2^ = 86%,*p* = 0.001, low certainty evidence), and venous thromboembolism (highest following general surgery; 9 studies, 4,796 patients, OR 1.75, 95%CI 1.25–2.45, I^2^ = 20.6%, *p* = 0.012, low certainty evidence). (See Supplementary Table 5).

### Meta-regression

A series of weighted univariable random-effects meta-regression analyses were performed to examine the relationship between unadjusted odds ratios and characteristics that may explain their variation. No study characteristic, including median year of study recruitment, study continent, cardiac versus non-cardiac surgery and overall study quality as assessed by the Newcastle Ottawa Scale (Supplementary Table 4), explained the observed heterogeneity in odds ratio for any of the non-fatal outcomes. However, several patient characteristics were predictive (Supplementary Figure 2A-2F). Firstly, meta-regression of the weighted mean age of each study on postoperative myocardial infarction and stroke demonstrated inverse linear relationships for both outcomes: slope − 0.05, 95% CI (− 0.09- -0.02) *p* < 0.001 for myocardial infarction, and slope − 0.04, 95% CI (− 0.08 - - 0.01) *p* = 0.031 for stroke. A similar inverse linear relationship was observed between the excess risk of myocardial infarction and prevalence of diabetes (slope − 0.02, 95% CI -0.03 - -0.01, *p* = 0.004), and also between stroke risk and prevalence of ischemic heart disease (slope − 0.02, 95% CI -0.04 - -0.01, *p* = 0.001); the latter relationship was maintained in multivariable meta-regression adjusted for age and diabetes mellitus (slope − 0.02, 95%CI -0.01- -0.01, *p* = 0.006).

An inverse linear relationship was also seen with the excess odds of pneumonia and age (slope − 0.04, 95% CI -0.06 – − 0.01, *p* = 0.003) and prevalence of diabetes (slope − 02, 95%CI -0.02 – 0.0, p = 0.003).

Univariable meta-regression identified a linear relationship between the risk of surgical site infections and prevalence of ischemic heart disease (slope 0.03, 95%CI 0.01–0.05, p = 0.006).

### Risk of Bias

Morbidity was inconsistently reported across studies: surgical site infection (37 studies) and sepsis (35 studies) were the most frequently reported. Seventeen of the 49 studies did not have explicit definitions of complications. Two studies [29, 60] reported post-operative complications graded by the Clavien-Dindo classification of surgical complications. Outcomes that were reported were of good quality, but comparability of patient groups on the basis of analysis was poor in 24 (49%) studies due to the absence of multivariable adjustment for patient demographics and co-morbidities [18–20, 22, 25–28, 32, 34, 35, 37–39, 41, 43, 46, 47, 49–51, 57, 59–61]. (Supplementary Table 4: Methodological quality of each study assessed by the NOS scale). Neither the funnel plot (Supplementary Figures 3A-E) nor Egger’s test (*p* = 0.328) suggested evidence of publication bias. Inter-rater variability between the two independent reviewers was strong (κ = 0.81).

The certainty in the quality of evidence was deemed to be low. The quality of evidence was downgraded both due to concerns with risk of bias (vide-supra), and for inconsistency due to residual heterogeneity. Having said that, the large magnitude of the odds estimate improved the strength of the evidence (Table S6).

## Discussion

This study demonstrated that patients with ESKD requiring chronic dialysis have increased odds of postoperative cardiovascular complications (myocardial infarction and stroke) and infectious complications (sepsis, surgical site infections and pneumonia) compared to patients with normal kidney function. This meta-analysis demonstrated a two- to fivefold increase in odds of postoperative myocardial infarction and stroke for patients on chronic dialysis, irrespective of surgical discipline. ESKD and dialysis are well established independent risk factors for adverse cardiovascular events; this relationship can be attributed to a number of non-traditional risk factors including inflammation, anaemia, calcium-phosphate imbalance and oxidative stress, in addition to dialysis specific factors such as intradialytic hypotension and myocardial stunning [64]. However, in studies that reported multivariable adjusted odds ratios, this excess risk was substantially attenuated. For instance, the odds of a postoperative stroke for chronic dialysis patient following general surgery was no different when compared to a patient with normal kidney function. Furthermore, meta-regression demonstrated that the excess odds of myocardial infarction attributable to receiving chronic dialysis treatment was less apparent with older age and in the presence of diabetes mellitus. A similar interaction was also observed between stroke risk, older age and presence of ischaemic heart disease. Taken together, these findings suggest that age and non-dialysis related comorbidities, including diabetes and ischemic heart disease, contribute significantly to the heightened odds observed, and dialysis while a risk factor for adverse outcomes is not itself the dominant driver of perioperative morbidity in this population. Currently, patients wait-listed for kidney transplantation undergo screening for coronary artery disease using non-invasive functional testing to identify occult disease and consider revascularization [64]. However, no such recommendations exist for patients on chronic dialysis considering elective surgery.

The odds of sepsis, surgical site infections and pneumonia, were also consistently elevated in patients on chronic dialysis compared to patients with normal kidney function across all surgical disciplines, with the exception of surgical site infections in general surgery and postoperative sepsis following urology/gyane surgery. The excess odds of sepsis among chronic dialysis patients remained elevated, even after adjusting for age and comorbidities, with the odds being more than twofold higher after cardiac and general surgery. The magnitude of the odds of pneumonia and surgical site infections for chronic dialysis patients was substantially reduced in adjusted analyses. Interestingly, meta-regression showed neither patient age nor the presence of diabetes mellitus appeared to modify the excess odds of sepsis or surgical site infections attributable to being on dialysis. These results differed to those for myocardial infarction and stroke, suggesting that the observed excess risk of infectious complications may be explained by the impaired immunity associated with ESKD and chronic dialysis [65]. The use of immunosuppression was reported in very few studies, such that determining their influence was not possible even though this is an important clinical consideration in patients with ESKD. Another potential reason for the elevated odds ratios seen in chronic dialysis patients may be related to definition of ESKD used in the various studies. A large number of studies were undertaken using data from registries such as the American College of Surgeons National Surgical Quality Improvement Program (ACS NSQIP), Vascular Quality Improvement Program and The Society of Thoracic Surgeons Database (STS), where dialysis dependency was defined as a patients requiring any form of dialysis within 2 weeks of surgery. Thereby potentially including patients with dialysis-dependent acute kidney injury in the study cohort. Patients with acute kidney injury carry substantially increased mortality and morbidity risk and therefore may have exaggerated the findings [66]. Studies also poorly reported dialysis treatment-related variables known to predispose chronic dialysis patients to infections, such as the presence of indwelling medical devices (central venous catheter, arteriovenous grafts and peritoneal dialysis catheters which provide an entry point for organisms) [67]. Furthermore, other important dialysis factors known to influence mortality and morbidity among dialysis patients in general, including dialysis vintage, dialysis modality and adequacy, were insufficiently reported [68, 69]. Therefore, the competing influences of these factors were not able to be evaluated. Indeed, more advanced disease or delayed surgery may also explain the observed differences. Data on other important surgical technique factors such as operational time and anesthesia type were also not available for analysis. It is also possible that variation between studies in the outcomes reported and definitions contributed to the differences in observed odds ratios.

In a prior meta-analysis, a sub-group analysis of 13 cohort studies involving 97,709 patients with normal kidney function and 27,501 with non-dialysis-requiring chronic kidney disease identified kidney dysfunction as an important, independent risk factor for composite postoperative cardiovascular events (arrhythmia, heart failure, angina, cardiac arrest, pulmonary oedema) following vascular and general surgery [70]. The results of our meta-analysis support these findings and extend them by demonstrating that the elevated postoperative odds of cardiovascular and infectious complications likely also apply to patients with kidney disease treated with dialysis.

### Limitations of analysis

To allow for greater generalisability, a comprehensive search strategy was used to identify a large number of dialysis patients across all elective surgical types. Despite attempts to adjust for potentitally confounding variables, such as age, indication bias with residual confounding could not be excluded. Although most studies reported sufficient details for the population to be included, many of the studies did not report potentially important confounding variables, such as primary kidney disease, dialysis modality and vintage, dialysis access type, residual kidney function or use of immunosuppression. Publication bias could also not be confidently ruled out, especially for those outcomes reported by only a few studies (e.g. thromboembolic complications). In addition, there was uncertainty about how well events were captured and reported. Therefore, the true frequency of postoperative complications in dialysis patients following major elective surgery is unknown. The absence of an objective and reproducible approach to classification of complications, such as the Clavien-Dindo classification, limited fair comparison of surgical outcomes between different patient populations and surgery types [9]. Finally, the selection of study cohorts was of good quality, but comparability of patient groups on the basis of analysis was poor in 24 (49%) studies due to the absence of multivariable adjustment for patient demographics and co-morbidities.

Future studies need to be more thorough in reporting patient baseline dialysis characteristics, procedural information and postoperative morbidity to allow for more informative analyses with adjustment for confounding. For instance, the microbiology of pathological organisms was not reported by any studies. Recovery of organisms from cultured specimens may not only inform future guidelines for empiric therapy in the perioperative setting, but also indicate potential sources. This is of particular importance given the higher odds of infectious complications demonstrated in this review [65]. Moreover, current perioperative risk assessments tools are unvalidated in chronic dialysis patients and fail to incorporate important dialysis-related characteristics that may potentially influence perioperative outcomes [71]. Research is needed to further risk stratify patients and facilitate intervention to mitigate perioperative cardiovascular and infectious complications.

## Supplementary Information

**Additional file 1: Table S1.** Search strategy to be used in EMBASE. **Table S2.** Search Strategy for MEDLINE. **Table S3.** Search strategy for CENTRAL. **Table S4.** Methodological quality of included studies (based on Newcastle-Ottawa scale). **Table S5.** Meta-analysis of non-fatal post-operative complications. **Table S6.** Post-operative morbidity outcomes and grading of the certainty of evidence using GRADE. Figure 1: Odds of postoperative pneumonia for chronic dialysis patients. Figure 2a-g: Meta-regression.

## Data Availability

The dataset used and analysed during the current study are available from the corresponding author on reasonable request.

## Abbreviations

CI: Confidence intervals
ESKD: End-stage kidney disease
GRADE: Grading of Recommendations, Assessment, Development and Evaluations
ICD: International classification of disease
NOS: Newcastle-Ottawa Scale
OR: Odds Ratio
