# Supplementary material for: Morbidity after elective surgery in patients on chronic dialysis: a systematic review and meta-analysis
Source: BMC Nephrol. 2021 Mar 18;22:97. doi: 10.1186/s12882-021-02279-0 (PMC7977605; doi:10.1186/s12882-021-02279-0)
Supplement: Supplementary file 1 — Additional file 1: Table S1. Search strategy to be used in EMBASE. Table S2. Search Strategy for MEDLINE. Table S3. Search strategy for CENTRAL. Table S4. Methodological quality of included studies (based on Newcastle-Ottawa scale). Table S5. Meta-analysis of non-fatal post-operative complications. Table S6. Post-operative morbidity outcomes and grading of the certainty of evidence using GRADE. Figure 1: Odds of postoperative pneumonia for chronic dialysis patients. Figure 2a-g: Meta-regression. [file 12882_2021_2279_MOESM1_ESM.docx]

**Morbidity after elective surgery in patients on chronic dialysis: a systematic review and meta-analysis**

Dharmenaan Palamuthusingam^1,2,3*^, Arun Nadarajah^4^, MBBS, David Wayne Johnson^5,6,7^, Elaine Marie Pascoe^8^, Carmel Marie Hawley^5,6,8^, Magid Fahim^5,8^

# Supplementary material (online):

**Supplementary Table 1: Search strategy to be used in EMBASE**

1. 'dialysis':de,ti,ab
2. 'end-stage renal disease':de,ti,ab
3. 'renal replacement therapy':de,ti,ab
4. 'hemodialysis':de,ti,ab
5. 'peritoneal dialysis':de,ti,ab
6. 1 OR 2 OR 3 OR 4 OR 5
7. 'continuous renal replacement therapy'/exp
8. 'continuous renal replacement therapy'
9. 'acute kidney failure'/exp
10. 'acute kidney failure'
11. 'kidney transplantation'/exp
12. 'kidney transplantation'
13. 7 OR 8 OR 9 OR 10 OR 11 OR 12 OR 13
14. 6 NOT 13
15. surgery:de,ti,ab
16. 'postoperative complication'/mj
17. 'cardiovascular mortality'/exp
18. 'hospital mortality'/exp
19. 'surgical mortality'/exp
20. 'anastomosis dehiscence'/exp
21. 'anastomosis leakage'/exp
22. 'failed back surgery syndrome'/exp
23. 'gastric band erosion'/exp
24. 'vein graft disease'/exp
25. 'paravalvular leak'/exp
26. 'postoperative edema'/exp
27. 'postoperative hemorrhage'/exp
28. 'postoperative ileus'/exp
29. 'postoperative infection'/exp
30. 'postoperative inflammation'/exp
31. 'postoperative thrombosis'/exp
32. 'periprosthetic fracture'/exp
33. 'prosthetic valve dysfunction'/exp
34. 'surgical infection'/exp
35. 'surgical injury'/exp
36. 'heart infarction'/exp
37. 'cerebrovascular accident'/exp
38. 'hospital readmission'/exp
39. 'reoperation'/exp
40. 'blood transfusion'
41. 16 OR 17 OR 18 OR 19 OR 20 OR 21 OR 22 OR 23 OR 24 OR 25 OR 26 OR 27 OR 28 OR 29 OR 30 OR 31 OR 32 OR 33 OR 34 OR 35 OR 36 OR 37 OR 38 OR 39 OR 40
42. 14 AND 15 AND 41

**Supplementary Table 2: Search Strategy for MEDLINE**

1. AB dialysis
2. TI dialysis
3. AB 'renal dialysis'
4. TI 'renal dialysis'
5. AB hemodialysis
6. TI hemodialysis
7. AB 'end stage renal disease'
8. AB 'end stage renal failure'
9. AB 'end stage kidney failure'
10. AB 'end stage kidney disease'
11. TI 'end stage renal disease'
12. TI 'end stage renal failure'
13. TI 'end stage kidney failure'
14. TI 'end stage kidney disease'
15. 1 OR 2 OR 3 OR 4 OR 5 OR 6 OR 7 OR 8 OR 9 OR 10 OR 11 OR 12 OR 13 OR 14
16. AB transplantation
17. TI transplantation
18. AB acute kidney injury
19. TI acute kidney injury
20. AB continous renal replacement therapy
21. TI continous renal replacement therapy
22. 16 OR 17 OR 18 OR 19 OR 20 OR 21 OR 22
23. 15 NOT 22
24. AB surgery
25. TI surgery
26. MM "Specialties, Surgical+"
27. 24 OR 25 OR 26
28. MM “Postoperative Complications”
29. MH "Cause of Death"
30. MH "Survival"
31. MH "Arrhythmias, Cardiac+"
32. MH "Death+"
33. MH "Hemorrhage+"
34. MH "Hypovolemia"
35. MH "Seroma"
36. MH "Serositis"
37. MH "Abscess"
38. MH "Cellulitis"
39. MH "Empyema"
40. MH "Systemic Inflammatory Response Syndrome+"
41. MH "Intraoperative Complications"
42. MH "Infarction"
43. MH "Fat Necrosis"
44. MH "Gangrene"
45. MH "Femur Head Necrosis"
46. MH "Anastomotic Leak"
47. MH "Graft Occlusion, Vascular"
48. MH "Incisional Hernia"
49. MH "Malignant Hyperthermia"
50. MH "Postcholecystectomy Syndrome"
51. MH "Postgastrectomy Syndromes"
52. MH "Postoperative Hemorrhage"
53. MH "Postpericardiotomy Syndrome"
54. MH "Prosthesis-Related Infections"
55. MH "Prosthesis Failure"
56. MH "Surgical Wound Dehiscence"
57. MH "Surgical Wound Infection"
58. MH "Respiratory Aspiration+"
59. MH "Multiple Organ Failure"
60. MH "Shock, Cardiogenic"
61. MH "Shock, Hemorrhagic"
62. MH "Ulcer"
63. MH "Arrhythmias, Cardiac"
64. MH "Death, Sudden, Cardiac"
65. MH "Out-of-Hospital Cardiac Arrest"
66. MH "Edema, Cardiac"
67. MH "Heart Failure, Diastolic"
68. MH "Heart Failure, Systolic"
69. MH "Acute Coronary Syndrome"
70. MH "Angina, Unstable+"
71. MH "Coronary Artery Disease"
72. MH "Coronary Occlusion"
73. MH "Anterior Wall Myocardial Infarction"
74. MH "Inferior Wall Myocardial Infarction"
75. MH "Non-ST Elevated Myocardial Infarction"
76. MH "Shock, Cardiogenic"
77. MH "ST Elevation Myocardial Infarction"
78. MH "Coronary Thrombosis"
79. MH "Postpericardiotomy Syndrome"
80. MH "Cerebral Infarction"
81. MH "Stroke, Lacunar"
82. MH "Brain Infarction"
83. MH "Pneumonia, Aspiration"
84. MH "Pneumonia, Bacterial"
85. MH "Pulmonary Atelectasis"
86. MH "Pulmonary Edema"
87. MH "Pulmonary Embolism"
88. MH "Length of Stay"
89. MH "Patient Readmission"
90. MH "Erythrocyte Transfusion"
91. MH "Blood Transfusion"
92. MH "Reoperation"
93. MH "Second-Look Surgery"
94. 28 OR 29 OR 30 OR 30 OR 31 OR 32 OR 33 OR 34 OR 35 OR 36 OR 37 OR 38 OR 39 40 OR 41 OR 42 OR 43 OR 44 OR 45 OR 46 OR 47 OR 48 OR 49 OR 50 OR 51 OR 52 OR 53 OR 54 OR 55 OR 56 OR 57 OR 58 OR 59 OR 60 OR 61 OR 62 OR 63 OR 64 OR 65 OR 66 OR 67 OR 68 OR 69 OR 70 OR 71 OR 72 OR 73 OR 74 OR 75 OR 76 OR 77 OR 78 OR 79 OR 80 OR 81 OR 82 OR 83 OR 84 OR 85 OR 86 OR 87 OR 88 OR 89 OR 90 OR 91 OR 92 OR 93
95. 23 AND 27 AND 94

**Supplementary Table 3: Search strategy for CENTRAL**

1. #1 dialysis
2. #2 end stage kidney disease
3. #3 end stage renal disease
4. #4 hemodialysis
5. #5 peritoneal dialysis
6. #6 (dialysis):ti,ab,kw OR (end stage renal disease):ti,ab,kw OR (end stage kidney disease):ti,ab,kw OR (haemodialysis):ti,ab,kw OR (peritoneal dialysis):ti,ab,kw (Word variations have been searched)
7. #7 surgery
8. #8 perioperative outcomes
9. #9 perioperative mortality
10. #10 perioperative complications
11. #11 postoperative complications
12. #12 postoperative mortality
13. #13 #8 OR #9 OR #10 OR #11 OR #12
14. #14 #6 AND #7 AND #13

**Supplementary Table 4: Methodological quality of included studies (based on Newcastle-Ottawa scale)**

| **First Author** | **Cohort Study Design** | **Dialysis Modality** | **Selection /🟑🟑🟑🟑** | **Comparability /🟑🟑** | **Outcomes /🟑🟑🟑** |
| --- | --- | --- | --- | --- | --- |
| Al Sarraf, 2011 | Retrospective | Unspecified | **🟑🟑🟑🟑** | **🟑🟑** | **🟑🟑🟑** |
| Ambur 2019 | Retrospective | Both | **🟑🟑🟑** | **🟑🟑** | **🟑🟑🟑** |
| Andalib, 2016 | Retrospective | Unspecified | **🟑🟑🟑🟑** | **-** | **🟑🟑🟑** |
| Balceniuk 2019 | Retrospective | Both | **🟑🟑🟑** | **🟑🟑** | **🟑🟑🟑** |
| Barbas, 2014 | Retrospective | Unspecified | **🟑🟑🟑** | **🟑🟑** | **🟑🟑🟑** |
| Cancienne 2019 | Retrospective | Both | **🟑🟑** | **-** | **🟑🟑** |
| Charytan, 2007 | Retrospective | Both | **🟑🟑🟑🟑** | **🟑🟑** | **🟑🟑🟑** |
| Cheng, 2013 | Retrospective | Both | **🟑🟑🟑** | **🟑🟑** | **🟑🟑🟑** |
| Chikuda, 2012 | Retrospective | Haemodialysis | **🟑🟑🟑** | **-** | **🟑🟑🟑** |
| Chikwe,2010 | Retrospective | Unspecified | **🟑🟑🟑🟑** | **🟑🟑** | **🟑🟑🟑** |
| Chung, 2017 | Retrospective | Unspecified | **🟑🟑🟑🟑** | **🟑🟑** | **🟑🟑🟑** |
| Cloyd, 2014 | Retrospective | Haemodialysis | **🟑🟑** | **🟑🟑** | **🟑🟑🟑** |
| Cooper, 2006 | Retrospective | Unspecified | **🟑🟑🟑🟑** | **🟑🟑** | **🟑🟑🟑** |
| Ekici, 2009 | Retrospective | Peritoneal Dialysis | **🟑🟑🟑🟑** | **-** | **🟑🟑🟑** |
| Fornara, 1998 | Retrospective | Haemodialysis | **🟑🟑🟑** | **-** | **🟑🟑🟑** |
| Fukushima, 2005 | Prospective | Haemodialysis | **🟑🟑🟑** | **-** | **🟑🟑🟑** |
| Gajdos, 2013 | Retrospective | Both | **🟑🟑🟑🟑** | **🟑🟑** | **🟑🟑🟑** |
| Gajdos, 2013 | Retrospective | Both | **🟑🟑🟑🟑** | **🟑🟑** | **🟑🟑🟑** |
| Griffin 2019 | Retrospective | Haemodialysis | **🟑🟑🟑🟑** | **-** | **🟑🟑🟑** |
| Hibino, 2016 | Retrospective | Haemodialysis | **🟑🟑🟑🟑** | **-** | **🟑🟑🟑** |
| Hickson, 2018 | Retrospective | Haemodialysis | **🟑🟑🟑🟑** | **-** | **🟑🟑🟑** |
| Hickson 2018 | Retrospective | Both | **🟑🟑🟑🟑** | **-** | **🟑🟑🟑** |
| Hu, 2015 | Retrospective | Unspecified | **🟑🟑🟑🟑** | **🟑🟑** | **🟑🟑🟑** |
| Inoue 2018 | Retrospective | Both | **🟑🟑🟑** | **-** | **🟑🟑🟑** |
| Kan, 2004 | Retrospective | Both | **🟑🟑🟑🟑** | **-** | **🟑🟑🟑** |
| Lantis 2001 | Retrospective | Unspecified | **🟑🟑🟑** | **-** | **🟑🟑🟑** |
| Lin 2019 | Retrospective | Unspecified | **🟑🟑🟑** | **🟑🟑** | **🟑🟑** |
| Lizaur-Utrilla, 2016 | Retrospective | Unspecified | **🟑🟑🟑🟑** | **-** | **🟑🟑🟑** |
| Marique, 2017 | Retrospective | Unspecified | **🟑🟑🟑🟑** | **🟑🟑** | **🟑🟑🟑** |
| May 2018 | Retrospective | Both | **🟑🟑🟑** | **-** | **🟑🟑🟑** |
| Montgomery 2019 | Retrospective | Unspecified | **🟑🟑🟑🟑** | **-** | **🟑🟑🟑** |
| Murai, 2007 | Retrospective | Haemodialysis | **🟑🟑🟑🟑** | **-** | **🟑🟑** |
| O Hare | Retrospective | Unspecified | **🟑🟑🟑🟑** | **🟑🟑** | **🟑🟑🟑** |
| Ottesen, 2018 | Retrospective | Unspecified | **🟑🟑🟑** | **🟑🟑** | **🟑🟑🟑** |
| Ottesen, 2018 | Retrospective | Unspecified | **🟑🟑🟑** | **🟑🟑** | **🟑🟑🟑** |
| Ponumsamy, 2015 | Retrospective | Unspecified | **🟑🟑🟑** | **🟑🟑** | **🟑🟑🟑** |
| Rahmanian, 2008 | Retrospective | Unspecified | **🟑🟑🟑** | **🟑🟑** | **🟑🟑🟑** |
| Rao, 2017 | Retrospective | Unspecified | **🟑🟑🟑** | **🟑🟑** | **🟑🟑🟑** |
| Rao 2014 | Retrospective | Unspecified | **🟑🟑🟑** | **-** | **🟑🟑** |
| Raza, 2017 | Retrospective | Haemodialysis | **🟑🟑🟑** | **-** | **🟑🟑** |
| Schmitges, 2012 | Retrospective | Unspecified | **🟑🟑🟑** | **🟑🟑** | **🟑🟑** |
| Schneider, 2009 | Retrospective | Unspecified | **🟑🟑🟑** | **-** | **🟑🟑** |
| Tam 2015 | Retrospective | Unspecified | **🟑🟑🟑🟑** | **🟑🟑** | **🟑🟑** |
| Thourani, 2012 | Retrospective | Haemodialysis | **🟑🟑🟑🟑** | **🟑🟑** | **🟑🟑🟑** |
| Vasileva 2014 | Retrospective | Unspecified | **🟑🟑🟑🟑** | **🟑🟑** | **🟑🟑🟑** |
| Wong 2003 | Retrospective | Unspecified | **🟑🟑🟑🟑** | **-** | **🟑🟑🟑** |
| Yamashita 2012 | Retrospective | Both | **🟑🟑🟑🟑** | **-** | **🟑🟑🟑** |
| Yamauchi, 2012 | Retrospective | Haemodialysis | **🟑🟑🟑🟑** | **-** | **🟑🟑🟑** |
| Yu 2011 | Prospective | Haemodialysis | **🟑🟑🟑🟑** | **-** | **🟑🟑🟑** |

**Selection criteria (out of 4 stars):** Representation of exposed cohort, selection of non-exposed group, ascertainment of exposure, demonstration that outcome of interest was not present at start of study

**Comparability (out of 2 stars):** Comparability of cohorts by the design or analysis

**Outcomes (out of 3 stars):** Assessment of outcomes, Follow-up duration and adequacy of follow-up of cohorts

**Supplementary Table 5: Meta-analysis of non-fatal post-operative complications**

|  | **Unadjusted outcomes** | | | | | | | | | **Adjusted ratios** | | | |
| --- | --- | --- | --- | --- | --- | --- | --- | --- | --- | --- | --- | --- | --- |
| **Outcome** | **Type of Surgery** | **Number of studies** | **Dialysis patients** | **Median rate (%)** | **Normal kidney function** | **Median rate (%)** | **Overall effect OR (95% CI)** | ***I*^2^(%)** | **P** | **Number of studies** | **Overall effect OR (95% CI)** ^c^ | ***I*^2^** | **P** |
| Return to theatre | Cardiac | 10 | 10 744 | 6.0 | 226 300 | 3.2 | 1.61 (1.12-2.30) | 85 | <0.001 | 1 | 2.10 (1.95-2.25) | - | - |
|  | General | 7 | 4 562 | 5.4 | 936 063 | 2.0 | 2.75 (1.98-3.81) | 83 | <0.001 | 3 | 1.83 (1.58-2.09) | 32 | 0.229 |
|  | Orthopaedic | 4 | 2 071 | 3.9 | 356 859 | 3.2 | 1.96 (0.63-6.06) | 95 | <0.001 | 3 | 1.97 (1.51-2.44) | 65 | 0.059 |
|  | Vascular | 4 | 3 603 | 16.6 | 74 911 | 8.8 | 2.13 (1.33-3.41) | 94 | <0.001 | 2 | 2.17 (1.87-2.48) | 90 | <0.001 |
|  | Urology/gynaecology | 0 | - | - | - | - | - | - | - | - | - | - | - |
| Transfusion requirement | Cardiac | 6 | 3 144 | 79.1 | 113 325 | 34.6 | 4.23 (2.80-6.37) | 87 | 0.000 | 2 | 0.70 (-.05-1.44) | 76 | 0.043 |
|  | General | 6 | 2 966 | 1.7 | 744 727 | 0.5 | 3.21 (2.14-4.81) | 56 | 0.046 | 1 | 1.78 (1.26-2.29) | - | - |
|  | Orthopaedic | 6 | 6 237 | 29.0 | 8 729 950 | 11.3 | 2.47 (1.84-3.32) | 92 | 0.000 | - | - | - | - |
|  | Vascular | 2 | 1 131 | 1.4-100 ^a^ | 11 730 | 0.4-89.4 ^a^ | 3.49 (1.96-6.21) | 0 | 0.768 | - | - | - | - |
|  | Urology/gynaecology | 3 | 1 505 | 12.1 | 12 517 | 5.8 | 2.16 (1.83-2.55) | 0 | 0.923 | 1 | 2.05 (1.54-2.56) | - | - |
| Thromboembolic events (DVT/PE) | Cardiac | 1 | 1 300 | 0.2 ^b^ | 18 387 | 0.2 | 1.15 (0.35-3.73) | - | - | - |  |  |  |
|  | General | 9 | 4 796 | 1.2 | 961 014 | 0.5 | 1.75 (1.25-2.45) | 21 | 0.260 | 1 | 0.80 (0.60-1.20) | - | - |
|  | Orthopaedic | 6 | 3 812 | 1.2 | 2 911 782 | 1.2 | 1.56 (0.84-2.89) | 69 | 0.012 | - | - | - | - |
|  | Vascular | 4 | 5 308 | 1.8 | 84 484 | 0.9 | 2.12 (0.56-8.07) | 97 | 0.000 | 1 | 1.46 (0.92-2.32) | - | - |
|  | Urology/gynaecology | 1 | 445 | 0.7 | 7870 | 0.8 | 0.87 (0.27-2.78) | - | - | - | - | - | - |

^a^Only 2 studies and therefore the complication rate from both studies are provided. If only a single study, then the incidence from that study is shown.

^b^ Single study

^c^In instances where only a single study is available, then no meta-analysis was performed.

**Supplementary Figure 1:** Odds of postoperative pneumonia for chronic dialysis patients

Author

Year

Chronic dialysis

Normal kidney function

Unadjusted OR

Adjusted OR


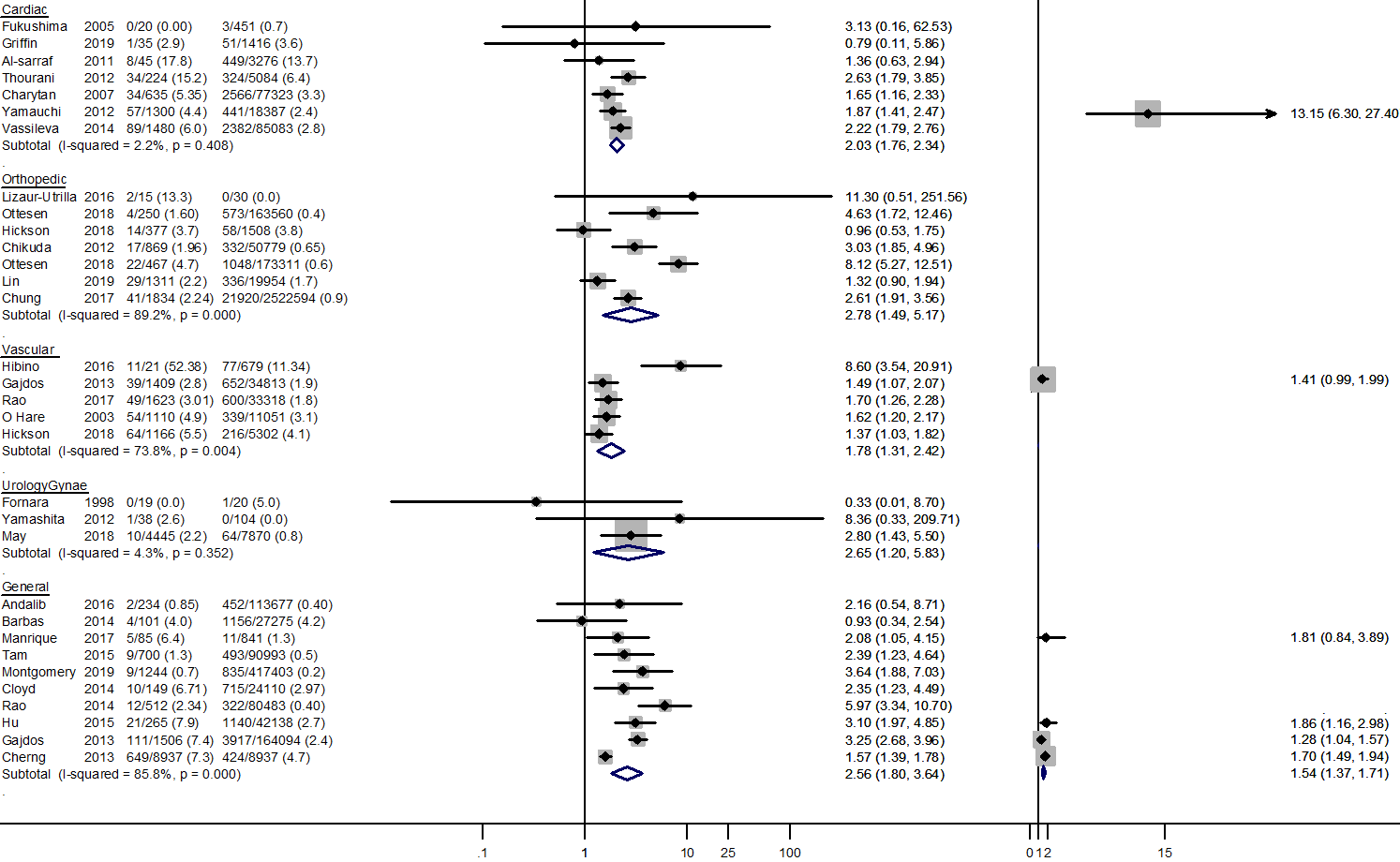


Higher odds on chronic dialysis

Lower odds on chronic dialysis

Higher odds on chronic dialysis

Lower odds on chronic dialysis

**Supplementary Figure 2A-G: Meta-regression**

**Figure A: Meta-regression for postoperative myocardial infarction odds by weighted mean age**

Each circle represents a study; the circle size is representative of the weight of that study in the analysis. The relation between logarithmic myocardial infarction odds ratio and the weighted mean age of dialysis and non-dialysis patients is significant (slope -0.05, 95% CI -0.09 – -0.02, *I^2^* 59.2%, adjusted R^2^ 49.6%, p=0.002).

**Figure B: Meta-regression for postoperative myocardial infarction odds by prevalence of diabetes mellitus**

Each circle represents a study; the circle size is representative of the weight of that study in the analysis. The relation between logarithmic myocardial infarction odds ratio and the prevalence of diabetes mellitus of dialysis and non-dialysis patients is significant (slope -0.02, 95% CI -0.03 – -0.01, *I^2^* 47.3%, adjusted R^2^ 60.3%, p=0.004).

**Figure C: Meta-regression for postoperative stroke odds ratio by weighted mean age**

Each circle represents a study; the circle size is representative of the weight of that study in the analysis. The relation between logarithmic stroke odds ratio and the weighted mean age of dialysis and non-dialysis patients is significant (slope -0.04, 95% CI -0.08 – -0.01, *I^2^* 91.4%, adjusted R^2^ 24.3%, p=0.031).

**Figure D: Meta-regression for postoperative stroke odds ratio by prevalence of ischemic heart disease**

Each circle represents a study; the circle size is representative of the weight of that study in the analysis. The relation between logarithmic stroke odds ratio and the prevalence of ischemic heart disease of dialysis and non-dialysis patients is significant (slope -0.02, 95% CI -0.04 – -0.01, *I^2^* 75.0%, adjusted R^2^ 42.8%, p=0.008).

**Figure E: Meta-regression for surgical site infection risk by prevalence of ischemic heart disease**

Each circle represents a study; the circle size is representative of the weight of that study in the analysis. The relation between logarithmic surgical site infection odds ratio and the prevalence of ischemic heart disease of dialysis and non-dialysis patients is significant (slope 0.03, 95% CI 0.01 – 0.05, *I^2^* 84.0%, adjusted R^2^ 54.1%, p=0.006).

**Figure F: Meta-regression for postoperative pneumonia odds ratio by weighted mean age.**

Each circle represents a study; the circle size is representative of the weight of that study in the analysis. The relation between logarithmic pneumonia odds ratio and the weighted mean of dialysis and non-dialysis patients is significant (slope -0.04, 95% CI -0.06 – 0.01, *I^2^* 73.0%, adjusted R^2^ 51.7%, p=0.003).

**Figure G: Meta-regression for postoperative pneumonia odds ratio by prevalence of diabetes mellitus.**

Each circle represents a study; the circle size is representative of the weight of that study in the analysis. The relation between logarithmic pneumonia odds ratio and the prevalence of diabetes in dialysis and non-dialysis patients is significant (slope -0.02, 95% CI -0.02 – 0.00, *I^2^* 66.8%, adjusted R^2^ 52.7%, p=0.003).

**Supplementary Figure 3A-E: Funnel plots**

**Supplementary Figure 3A: Myocardial infarction**

**Supplementary Figure 3B: Stroke**

**Supplementary Figure 3C: Sepsis**

**Supplementary Figure 3D: Surgical Site Infection**

**Supplementary Figure 3E**

**Supplementary Table 6:** Post-operative morbidity outcomes and grading of the certainty of evidence using GRADE.

| **Outcomes (Studies)** | **Certainty of evidence (GRADE)** | **Rationale** | | | | | | |
| --- | --- | --- | --- | --- | --- | --- | --- | --- |
|  |  | **Risk of bias** | **Inconsistency** | **Indirectness** | **Imprecision** | **Publication bias** | **Strong association** | **Other Comments** |
| Myocardial infarction (31) | Low ^a, c, d, f^ | ↓ | – | – | ↓ | – | ↑ | Certainty of evidence not down-graded for inconsistency due to heterogeneity being explained by increasing patient age and prevalence of diabetes. |
| Stroke (35) | Low ^a, c, e, f^ | ↓ | – | – | – | – | ↑ | Certainty of evidence not down-graded for inconsistency due to heterogeneity being explained by increasing patient age and prevalence of ischemic heart disease. |
| Sepsis (35) | Low ^a, b, c, e, f^ | ↓ | ↓ | – | – | – | ↑ | – |
| Surgical site infection (37) | Low ^a, c, e, f^ | ↓ | ↓ | – | – | – | ↑ | Certainty of evidence not down-graded for inconsistency due to heterogeneity being explained by increasing prevalence of ischemic heart disease. |
| Pneumonia (32) | Low ^a, b, c, d, f^ | ↓ | ↓↓ | – | – | – | ↑ | – |

^a^ Certainty of evidence down-graded due to concerns of poor comparability between study cohorts

^b^ Certainty of evidence down-graded for inconsistency due to residual heterogeneity that remained unexplained despite investigators efforts to identify plausible explanation

^c^ Certainty of evidence not down-graded for indirectness

^d^ Certainty of evidence down-graded for imprecision, due to few events and wide confidence intervals.

^e^ Certainty of evidence not down-graded for imprecision due to significant number of events in both groups.

^f^ Certainty of evidence upgraded by one level due to large (>2 fold) risk estimate
